# Rapid Determination of 9 Tyrosine Kinase Inhibitors for the Treatment of Hepatocellular Carcinoma in Human Plasma by QuEChERS-UPLC-MS/MS

**DOI:** 10.3389/fphar.2022.920436

**Published:** 2022-06-21

**Authors:** Wen Jiang, Tingting Zhao, Xiaolan Zhen, Chengcheng Jin, Hui Li, Jing Ha

**Affiliations:** ^1^ College of Chemistry and Pharmaceutical Engineering, Hebei University of Science and Technology, Shijiazhuang, China; ^2^ College of Pharmacy, Hebei Medical University, Shijiazhuang, China; ^3^ Hebei Institute of Drug and Medical Device Inspection, Shijiazhuang, China

**Keywords:** tyrosine kinase inhibitors, UPLC-MS/MS, QuEChERS, plasma, hepatocellular carcinoma

## Abstract

A reliable and rapid method employing QuEChERS (Quick, Easy, Cheap, Effective, Rugged, and Safe) pretreatment coupled with ultra-performance liquid chromatography–tandem mass spectrometry (UPLC–MS/MS) was successfully developed and validated for the analysis of nine tyrosine kinase inhibitors (TKIs) in human plasma. Biological samples were extracted with acetonitrile and salted out with 350 mg of anhydrous magnesium sulfate (MgSO_4_), followed by purification with 40 mg of ethyl enediamine-N-propylsilane (PSA) adsorbents. All analytes and internal standards (IS) were separated on the Hypersil GOLD VANQUISH C18 (2.1 mm × 100 mm, 1.9 μM) column using the mobile phases composed of acetonitrile (phase A) and 0.1% formic acid in water (phase B) for 8.0 min. Detection was performed by selection reaction monitoring (SRM) in the positive ion electrospray mode. Lenvatinib, sorafenib, cabozantinib, apatinib, gefitinib, regorafenib, and anlotinib rendered good linearity over the range of 0.1–10 ng/ml, and 1–100 ng/ml for tivantinib and galunisertib. All linear correlation coefficients for all standard curves were ≥ 0.9966. The limits of detection (LOD) and the limits of quantitation (LOQ) ranged from 0.003 to 0.11 ng/ml and 0.01–0.37 ng/ml, respectively. The method was deemed satisfactory with an accuracy of -7.34–6.64%, selectivity, matrix effect (ME) of 90.48–107.77%, recovery, and stability. The proposed method is simple, efficient, reliable, and applicable for the detection of TKIs in human plasma samples as well as for providing a reference for the clinical adjustment of drug administration regimen by monitoring the drug concentrations in the plasma of patients.

## 1 Introduction

Cancer is a major public health issue across the world, with the associated global burden increasing dramatically owing to the aging of the population, environmental degradation, and undesirable lifestyle behaviors such as smoking and alcoholism (Noorolyai et al., 2019; Wu et al., 2019; Bray et al., 2020). Hepatocellular carcinoma (HCC) is a common malignant tumor with insidious onset, rapid progression, early recurrence, and poor prognosis, as well as consistently high rates of incidence and mortality (Dutta and Mahato, 2017).

Tyrosine kinase inhibitors (TKIs) are small molecule-targeted drugs that target receptor tyrosine kinases. Their action mechanism is based on competing with adenosine triphosphate (ATP) for binding to the ATP-binding site of the kinase domain in order to block or reduce the phosphorylation of tyrosine kinase and, ultimately, exert anti-tumor effects (Cammarota et al., 2022; Yang et al., 2022; Yao et al., 2022). TKIs are widely used in the treatment of small-cell lung cancer (SCLC) (Hwang et al., 2021), non-small cell lung cancer (NSCLC) (Lin et al., 2022; Ten et al., 2022), gastrointestinal mesenchymal tumor (GIST) (Mohammadi and Gelderblom, 2021; Foo et al., 2022; Klug et al., 2022), hepatocellular liver cancer (HCC) (Decraecker et al., 2021), renal cancer (RCC) (Fogli et al., 2020; Pedersen et al., 2021), and other cancers owing to their high selectivity and low adverse effects when compared with those of the traditional cytotoxic anticancer drugs (Xing et al., 2021). Sorafenib is the first first-line oral small molecule TKI approved by the U.S. Food and Drug Administration (FDA) for HCC, ushering in a new era of molecular targeting in HCC (Di et al., 2013; Decraecker et al., 2021). Sorafenib has been followed by other targeted drug studies in search of breakthroughs in molecularly targeted drug therapy for HCC. Currently, the first-line targeted agents include sorafenib and lenvatinib, while the second-line targeted agents for HCC include regorafenib and cabozantinib (Zhao et al., 2020; El-Khoueiry et al., 2021). In addition, results from a randomized, placebo-controlled, double-blind phase-III study of apatinib as the second-line treatment of Chinese patients with advanced HCC demonstrated that apatinib could significantly prolong the survival time of first-line resistant patients with advanced HCC and that it was well tolerated by patients in a safe and manageable manner (Qin et al., 2020; Qin et al., 2021). Tivantinib has been reported to downregulate the MET activity and the expression of downstream signaling pathways in tumor biopsy specimens (Rimassa et al., 2018). The safety and efficacy of galunisertib in combination with sorafenib have been reported in several publications (Yingling et al., 2018; Wick et al., 2020). In addition, it has been reported that the combination of gefitinib treatment for patients with intermediate to advanced HCC who failed to respond to lenvatinib treatment could effectively inhibit the progression of HCC (Jin et al., 2021), while anlotinib has also been demonstrated to be effective in the treatment of intermediate to advanced HCC (Guo et al., 2020). Therefore, all of the nine TKIs mentioned earlier exhibited anti-hepatocellular carcinogenic effects.

A recent review suggested that the specific metabolism (supporting the therapeutic schedule of 3 weeks on and 1 week off/month) of regorafenib may affect the blood levels and therapeutic efficacy (Granito et al., 2021). Patients with HCC may suffer from adverse events or serious adverse events associated with drug therapy owing to drug resistance and drug toxicity. An overview of the study on lenvatinib reported that 82% of patients in the trial reduced their dose or stopped treatment because of adverse effects (Rehman et al., 2021). Patients with mild or moderate renal and hepatic impairment may need to be closely monitored, according to a report of cabozantinib (D'Angelo et al., 2020). It is recommended that tivantinib requires monitoring of therapeutic agents in order to adjust the administered dose on time (Maharati et al., 2022). Therefore, monitoring the above nine TKIs have a significant meaning to improve drug efficacy and safety. It is necessary to develop a reliable, rapid, and sensitive method to monitor the concentration of anti-HCC drugs in order to facilitate clinical medication guidance.

Currently, the analysis of small-molecule tyrosinase inhibitors is mostly performed by liquid chromatography coupled with mass spectrometry (LC-MS), albeit the assay requires a good matrix effect (ME) of the sample. Accordingly, a suitable pretreatment technique needs to be selected to improve the purification efficiency and reduce the effect of the impurities. Based on the detection of TKIs, the commonly used pretreatment techniques include protein precipitation (PP) (Tibben et al., 2019; Ferrer et al., 2020; Iacuzzi et al., 2020; Krens et al., 2020; Aghai et al., 2021), liquid-liquid extraction (LLE) (Ni et al., 2017; Guan et al., 2019; Ezzeldin et al., 2020), salinization-assisted liquid-liquid extraction (SALLE) (Zhou et al., 2021), and solid-liquid extraction (SLE) (Sueshige et al., 2021), among others. While QuEChERS is an emerging pretreatment technique derived from dispersive solid-phase extraction (dSPE), it was initially applied in the field of pesticide residues. Recently, it was applied to the analysis of metabolites and other compounds in biological matrices, such as plasma and urine in parallel with the advancements in this technique.

In this study, we developed and validated the UPLC-MS/MS method combined with an emerging preprocessing technology QuEChERS (Quick, Easy, Cheap, Effective, Rugged, and Safe) for the detection of nine anti-HCC TKIs, including lenvatinib (LEN), sorafenib (SOR), cabozantinib (CBZ), apatinib (APA), gefitinib (GEF), regorafenib (RGF), anlotinib (ANL), tivantinib (TIV), and galunisertib (GAL).

## 2 Materials and Methods

### 2.1 Chemicals and Reagents

A total of nine TKIs standard substances were purchased from the Shanghai Yuanye Biotechnology Co., Ltd. (Shanghai, China). Propranolol was supplied by the China National Institute for China Drug and Biological Products Control. HPLC-grade methanol (MeOH), ethyl acetate (EtOAc), and acetone (CP) were purchased from Merck Drugs & Biotechnology (Darmstadt, Germany). HPLC-grade formic acid (FA) and acetic acid (HOAc) were purchased from Dikma (Beijing, China); LC/MS-grade acetonitrile (ACN) was acquired from ThermoFisher Scientific (Shanghai, China). Analytical-grade anhydrous magnesium sulfate (MgSO_4_) was purchased from the Tianjin Damao Chemical Reagent Factory (Tianjin, China). Octadecyl bonded silicagel (C18), florisil adsorbents, and graphitized carbon black (GCB) were supplied by Agela Technologies (Tianjin, China). NH_2_ and ethyl enediamine-N-propylsilane (PSA) were purchased from Agilent Technologies (Shanghai, China).

### 2.2 Instrument

Vanquish Flex Ultra-performance Liquid Chromatography (UPLC) and TSQ Altis Triple Quadrupole Mass Spectrometer (MS) (Thermo Fisher Scientific, United States), nitrogen blowing concentrator (Beijing Politech Instrument Co., Ltd., China), High-speed Refrigerated Centrifuge (Yancheng Kait Experimental Equipment Co., Ltd. China), Vortex Meter (Wiggens, Germany), Milli-Q Purification System (Millipore, United States), KQ-500E Ultrasonic Cleaner (Kunshan Ultrasonic Instrument Co., Ltd. China), and Electronic analytical balance (Mettler Toledo, United States).

### 2.3 Mass Conditions

The MS was operated in the positive ionization mode with electrospray ionization (ESI) and selection reaction monitoring (SRM) to analyze all compounds. Xcalibur software (Thermo Fisher Scientific) was applicated for data acquisition and processing. The ion source parameters were set as follows: the ionspray voltage was 3500 V, the sheath gas was 45 Arb, the aux gas was 10 Arb, the ion transfer tube temperature was 350°C, and the vaporizer temperature was 400°C. Argon at a pressure of 1.5 mTorr as collision gas for collision-induced dissociation (CID). In this method, the dwell time for per transition was 100 ms. The precursor ions and product ions of each compound, the fragmentor voltage, collision energy, and retention time are displayed in Table 1.

**TABLE 1:** The MS condition of the 9 tyrosine kinase inhibitors and IS.

Compounds	Parent (m/z)	Product (m/z)	Fragmentor voltage (V)	Collision voltage (eV)	Retention time (min)
LEN	427.1	370.1^a^/312.0	102	27/43	2.61
SOR	465.1	252.1^a^/270.1	113	33/24	3.67
CBZ	502.2	323.1^a^/297.1	139	37/35	3.10
RGF	483.1	270.1^a^/288.1	123	33/24	3.72
APA	398.2	212.1^a^/184.1	81	26/37	3.05
GEF	447.1	128.1^a^/100.1	75	24/47	2.74
ANL	408.2	339.1^a^/304.1	60	18/40	2.78
TIV	370.1	253.1^a^/158.1	65	21/22	3.46
GAL	370.1	336.1^a^/325.1	74	30/29	1.30
PRO	260.1	116.13^a^/183.1	53	18/18	3.05

a Quantification ion.

### 2.4 Chromatographic Conditions

Separation and analysis were achieved using the Hypersil GOLD VANQUISH C18 column (100 μM × 2.1 μM; 1.9 μM), and the mobile phase was acetonitrile (phase A) and 0.1% formic acid in water (phase B). The gradient elution was performed as follows: for the first 0.5 min, the mobile phase B was 85%, then the proportion of mobile phase B was decreased from 85 to 5% at 0.5–2 min and held for 4 min, the mobile phase B was restored to 85% within 1 min, and then equilibrated for 1 min in the final. The analytical runtime was 8 min, the flow rate was 0.3 ml/min, and the injection volume for analysis was 5 μL.

### 2.5 Stock Solutions, Working Solutions, and Quality Control Samples

The stock solutions of sorafenib, cabozantinib, gefitinib, regorafenib, anlotinib, tivantinib, galunisertib (1 mg/ml), lenvatinib (800 μg/ml), and IS (500 μg/ml) were prepared in methanol at room temperature and maintained at -20°C until further use.

Different concentrations of the mixing working stock solutions (100, 10, and 1 µg/ml) were prepared by dilution of the stock solutions in methanol and stored at −20°C until use. The mixed working solutions were diluted with methanol in a certain proportion to prepare a series of calibration curve samples, ranging in concentration from 0.1 to 100 ng/ml (LEN, SOR, CBZ, RGF, APA, GEF, ANL: 0.1, 0.2, 0.5, 1, 2, 5, 8, and 10 ng/ml; TIV, GAL: 1, 2, 5, 10, 20, 50, 80, and 100 ng/ml). The concentration of the final IS solution was 5 ng/ml.

Blank human plasma (200 μL) was spiked with a certain concentration of the mixed working solution (10 μL) in order to obtain the quality control (QC) samples. The low QC (LQC), the medium QC (MQC), and the high QC (HQC) samples were set at 0.2, 5, and 10 ng/ml concentration (TIV and GAL) and at 2, 50, and 100 ng/ml concentration (LEN, SOR, CBZ, RGF, APA, GEF, and ANL), respectively. All working solutions were stored in a polypropylene (PP) centrifuge tube at −20°C until further analyses.

### 2.6 Sample Preparation

The blood of healthy people and hepatocellular carcinoma patients were obtained from the Bethune International Peace Hospital (Hebei, China) to serve as a control.

The received blood samples were centrifuged for 10 min at 3,500 × g at 4°C, and the supernatant was separated in a PP centrifuge tube and stored at −20°C. The plasma was removed before use and then thawed at room temperature. After vortex for 1 min, 200 μL of the plasma sample was added to a 4-ml centrifuge tube, to which 10 μL of the 5 ng/ml IS solution was added and mixed for 10 s. Subsequently, the samples were added to 1.5 ml of acetonitrile for extraction and vortexed for 30 s. Then, 350 mg of the MgSO_4_ and 40 mg of the PSA were added respectively for salting out and purification. After vortexing for 30 s, the samples were centrifuged at 12,000 × g for 10 min. Then, 1 ml of the supernatant was filtered through a 0.22-µM microporous membrane and transferred into a centrifugal tube, followed by blowing with nitrogen at room temperature until dry. In the final step, the dried extracts were redissolved in 200 μL of the methanol solution and 5 μL of the final solution was injected into the UPLC-MS/MS system.

### 2.7 Method Validation

The established methods were validated in terms of selectivity, linearity, precision, accuracy, stability, and MEs based on the bioanalytical method validation guidelines by the US Food and Drug Administration (FDA) (U.S., 2018).

#### 2.7.1 Selectivity

The selectivity of the method was evaluated by analyzing six blank blood samples from diverse individuals and six lower limits of quantitation (LLOQ) samples. The resulting chromatograms were compared and the response of interfering components was set to < 20% of the response of the analytes and < 5% of the response of IS (Verougstraete et al., 2021).

#### 2.7.2 Calibration Curve and Lower Limit of the Quantification

Standard samples in different concentration ranges were obtained following “sample preparation” as detailed in Section 2.6. The concentration of the analytes was set as the horizontal coordinate (X) and the peak area ratio of the analytes to the internal standard as the vertical coordinate (Y). Then the calibration curves were assessed by a linearly weighted (1/x^2^) least-squares linear regression analysis (Fresnais et al., 2020). The correlation coefficient (R^2^) was > 0.990. The concentration of the calibration standard samples were based on the calibration curve, each calibration level was set to within ±15% of the nominal value, and the LLOQ was accepted for a ±20% range (He et al., 2017).

The limit of detection (LODs) and the limit of quantitation (LOQs) of the instrument was defined by the S/N (signal-to-noise ratio) (Kocan et al., 2018). The S/N of LODs was set to ≥ 3 and the S/N of LOQs to ≥ 10.

#### 2.7.3 Precision and Accuracy

Intra-day precision and accuracy of the method were obtained by analyzing the QC samples at three different levels (i.e., LQC, MQC, and HQC) and the LLOQ samples using six replicates on the same day. The inter-day accuracy and precision were evaluated on three consecutive days (Ferrer et al., 2020). Precision was expressed in terms of relative standard deviation (RSD) and accuracy in terms of relative error (RE). For each QC level, the RSD value was required to be < 15% and the deviation of the RE value was set to be within ±15%. For the LLOQ, the RSD was set to < 20% and RE within ±20% (Reis et al., 2018).

#### 2.7.4 Recovery and MEs

To ensure efficient recovery, six replicates of the three analyte concentration levels (i.e., LQC, MQC, and HQC) and one IS concentration level (5 ng/ml) were assessed. The analytical results of the blank plasma spiked with analyte after extraction (A) were compared to the samples spiked with the analyte before extraction (B). The extent of recovery in this experiment was evaluated based on the ratio of the analytes’ peak area to the IS area: (A/B) × 100% (Aghai et al., 2021).

The ratio of the peak area to the internal standard area was compared with QC samples (C) and the matrix-free samples(D). The ME of the QuEChERS-UPLC-MS/MS method was calculated as follows: C/D × 100%, and the RSD was set to be < 15% (Ogawa-Morita et al., 2017; Zheng et al., 2021).

#### 2.7.5 Stability

Stability assessments under different conditions were conducted for the QC (low- and high-concentration) samples, namely at room temperature (25°C) for 24 h, under refrigeration (4°C) for 48 h, in an autosampler for 72 h, under −20°C for 15 days, and in three different freeze-thaw cycles (−20°Cto room temperature); three parallel samples were set for each concentration and then tested. If the RSD was < 15%, stability was considered to be acceptable.

## 3 Results and Discussion

### 3.1 Comparison of the Preprocessing Methods

**FIGURE 1:**
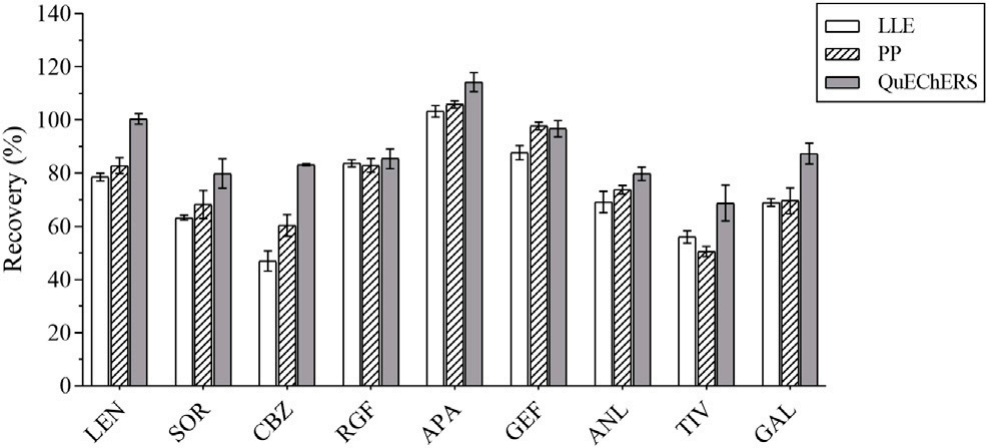
The recovery results of all analytes by the 3 pretreatment methods.

At the same spiked level, the effects of two frequently used pretreatment methods, that is, LLE and PP, and the emerging pre-treatment method QuEChERS on analyte recovery were evaluated. The recovery of the analytes in these three pretreatment methods is illustrated in Figure 1.

The QuEChERS purification method was performed according to the “sample preparation” method, as detailed in Section 2.6. Pure acetonitrile was used for PP. The IS solution (10 μL of 5 ng/ml) and 10 μL of the standard solution were added to the blank plasma sample and mixed for 30 s, followed by the use of 1 ml acetonitrile for PP. These results revealed that the acetonitrile PP method was efficient and convenient, although its purification effect was not ideal. Methyl tert-butyl ether was used for LLE, and 10 μL of the IS solution (5 ng/ml) and 10 μL of the standard solution were added to the blank plasma sample and mixed for 30 s, after which 1 ml of methyl tert-butyl ether was added for LLE. The recovery rates of several methods ranged from 47.85 to 95.01%. For instance, when compared with LLE and acetonitrile PP, the QuEchERs method revealed a higher recovery rate for most analytes, just as for the plasma samples used in this study. Accordingly, the QuEchERs method was selected for the pretreatment of plasma samples in this study.

### 3.2 Optimization of Preprocessing Methods

#### 3.2.1 Optimization of the Extraction Conditions

**FIGURE 2:**
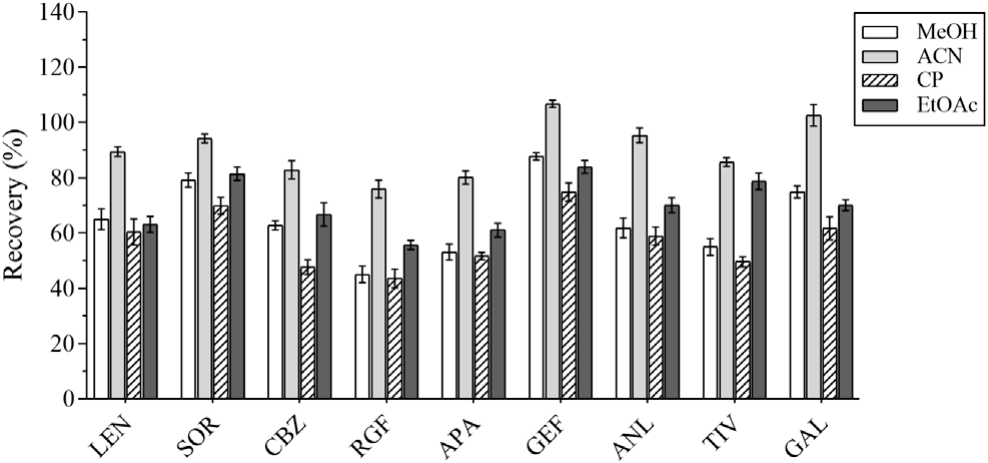
The recovery results of all analytes for different extraction solvents.

**FIGURE 3:**
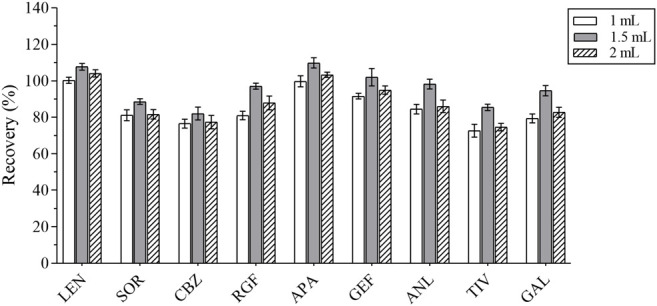
The recovery results of all analytes with different volumes of acetonitrile.

In the first place, the varieties and amounts of extraction solvents were optimized. Methanol, acetonitrile, ethyl acetate, and acetone were the commonly used extraction solvents (Perestrelo et al., 2019; Kanu et al., 2021; Chen et al., 2021). The recovery results were applied to evaluate the extraction effect of several organic solvents. Equal amounts of the intended analytes and 1 ml of the abovementioned organic solvents were then added; the experimental results revealed that the peak shape and the extraction effect of acetonitrile were better than that of the other solvents; the recovery results are shown in Figure 2. The extraction efficiency of this method was affected by the amount of extraction solvent and the addition of different volumes of acetonitrile (i.e., 1, 1.5, and 2 ml) respectively. The results are presented in Figure 3. The best extraction effect was achieved with the addition of 1.5 ml acetonitrile. Therefore, 1.5 ml of acetonitrile was selected for the extraction of these TKIs.

#### 3.2.2 Optimization of Salting-Out Conditions

**FIGURE 4:**
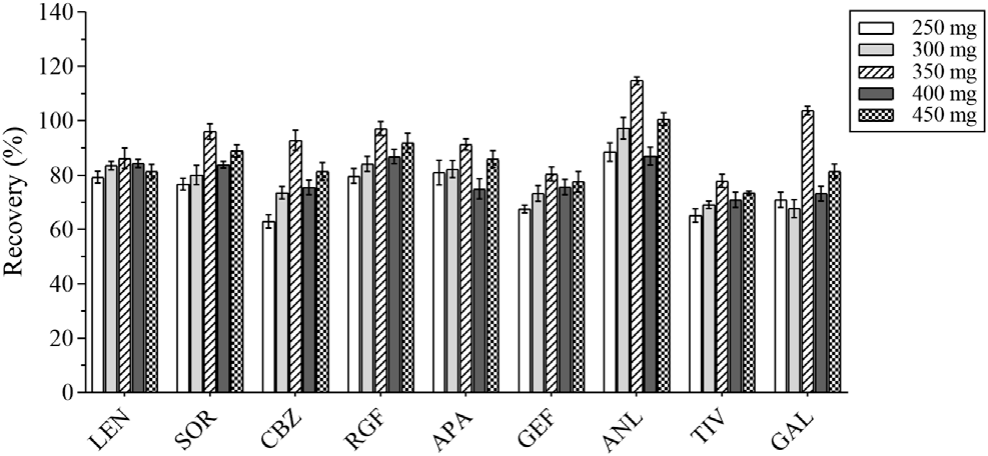
The recovery results of all analytes with different masses of anhydrous magnesium sulfate.

The composition of the human plasma matrix is complex and majorly attributable to water, which affects the determination of analytes and causes unnecessary loss to the MS. In this study, anhydrous MgSO_4_ was selected as the salting-out agent, and its dosage was optimized; the recovery of each analyte served as the evaluation index. Different masses of anhydrous MgSO_4_ (i.e., 250, 300, 350, 400, and 450 mg) were added for the experiments under the same spiking level (Figure 4). The best recovery of each analyte was obtained with 350 mg of anhydrous MgSO_4_.

#### 3.2.3 Optimization of the Adsorption Conditions

**FIGURE 5:**
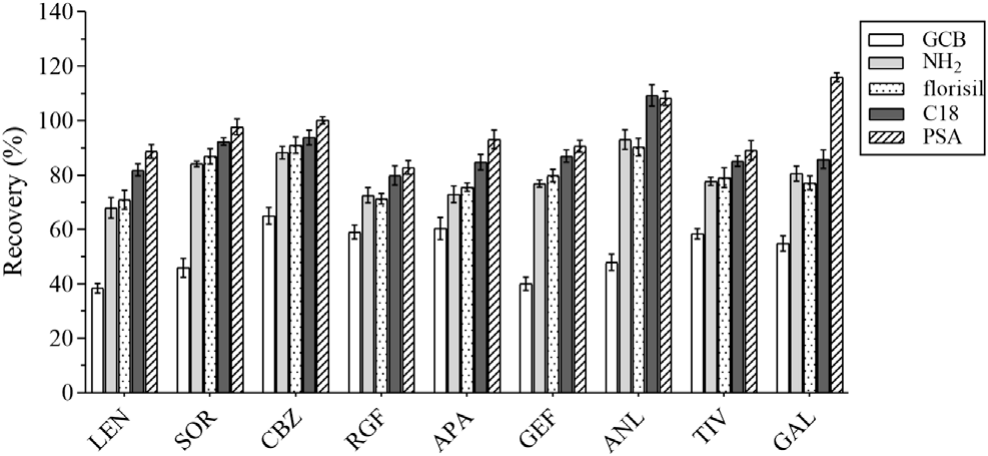
The recovery results of all analytes with 5 adsorbents.

**FIGURE 6:**
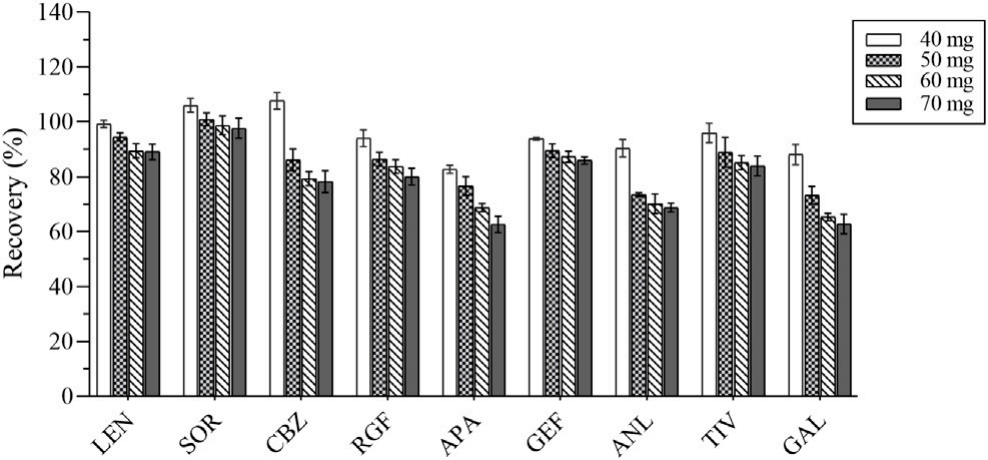
The recovery results of all analytes with different amounts of PSA.

The composition of the plasma is complex, including proteins, lipids, inorganic salts, and amino acids, all of which can influence the MEs. In this study, five adsorbents were selected, including ethyl enediamine-N-propylsilane (PSA), florisil, octadecyl bonded silica gel (C18), NH_2_, and graphitized carbon black (GCB). Their results were compared based on the recovery rate (Figure 5). GCB exhibited poor purification results outcome not deemed suitable for plasma purification. In contrast, PSA exhibited the best results and was hence recommended for the removal of sugars, fatty acids, and organic acids (Niu et al., 2020). Various amounts of PSA (i.e., 40, 50, 60, and 70 mg) were selected as the optimum amount of the salting-out agent for this assay. The experimental results were compared by recovery and are shown in Figure 6. Therefore, 40 mg of PSA was selected as the optimal amount of adsorbent in the QuEChERS pretreatment method.

### 3.3 Optimization of the Mass Spectrometric

**FIGURE 7:**
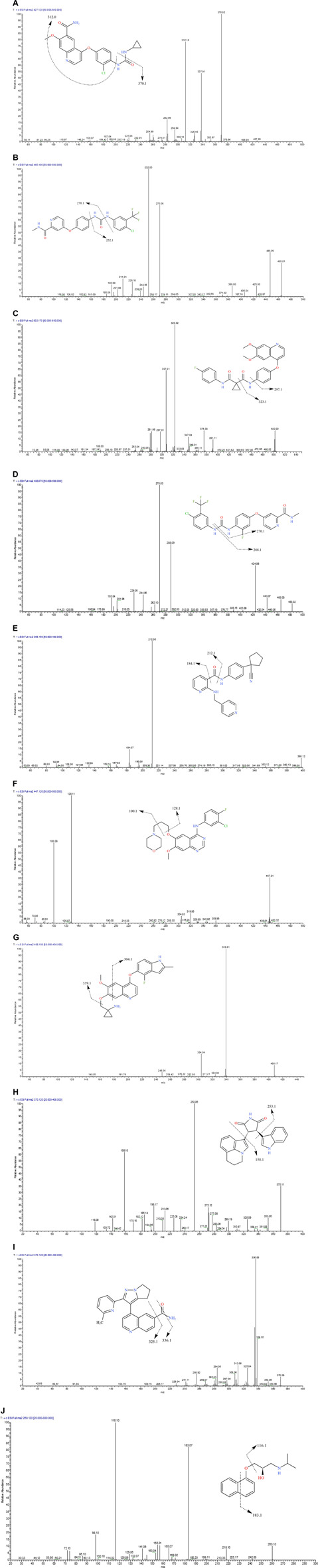
The product ion spectra of [M+H]^+^ of lenvatinib **(A)**, sorafenib **(B)**, cabozantinib **(C)**, regorafenib **(D)**, apatinib **(E)**, gefitinib **(F)**, anlotinib **(G)**, tivantinib **(H)**, galunisertib **(I)** and IS **(J)**, and their chemical structures.

All analytes and IS (1 μg/ml) were directly injected via a needle pump and scanned in the positive ion mode and negative ion mode. We found that the response of each substance was higher in the positive ion mode. Accordingly, nine TKIs were analyzed in the positive ion mode. One quantitative ion and one qualitative ion were selected for each substance. The product spectra and the proposed fragmentation patterns of each analyte and IS are depicted in Figure 7.

### 3.4 Optimization of the Chromatographic Conditions

In this experiment, we attempted to compare the separation performance of 4 columns, which included the Agilent Poroshell 120 EC-C18 (3.0 mm × 50 mm, 2.7 μM) column, Hypersil GOLD VANQUISH C18 (2.1 mm × 100 mm, 1.9 μM) column, the Agilent Eclipse Plus C18 (3.0 mm × 100 mm, 1.8 μM) column, and Agilent Eclipse Plus C18 (4.6 mm × 100 mm, 3.5 μM) column. The columns were evaluated under moderate concentrations of QC samples. The results revealed that the Agilent Poroshell 120 EC-C18 column gave a good peak shape, but a relatively concentrated peak time. The Hypersil GOLD VANQUISH C18 columns were found to be good for the analysis of each determinant, with good separation performance and peak shape. The Agilent Eclipse Plus C18 (3.0 mm × 100 mm, 1.8 μM) column could not separate the analytes adequately owing to the small difference in the polarity of the analytes, resulting in poor peak shape. On the other hand, the Agilent Eclipse Plus C18 (4.6 mm × 100 mm, 3.5 μM) column was longer, and the analytes were slower to the peak. According to the experimental results, the Hypersil GOLD VANQUISH C18 column was selected for use as the analytical column in this experiment.

Moreover, the effects of different combinations of organic and aqueous phases on the peak shapes and response values of the analytes were investigated. Methanol and acetonitrile were examined and the response of the analytes was found to be slightly higher with acetonitrile as the organic phase. Subsequently, different aqueous phases were examined (e.g., water, 0.1% formic acid with water, and 0.1% acetic acid with water), and the results revealed that the optimal mobile phase was 0.1% formic acid water and acetonitrile.

### 3.5 Method Validation

#### 3.5.1 Selectivity

**FIGURE 8:**
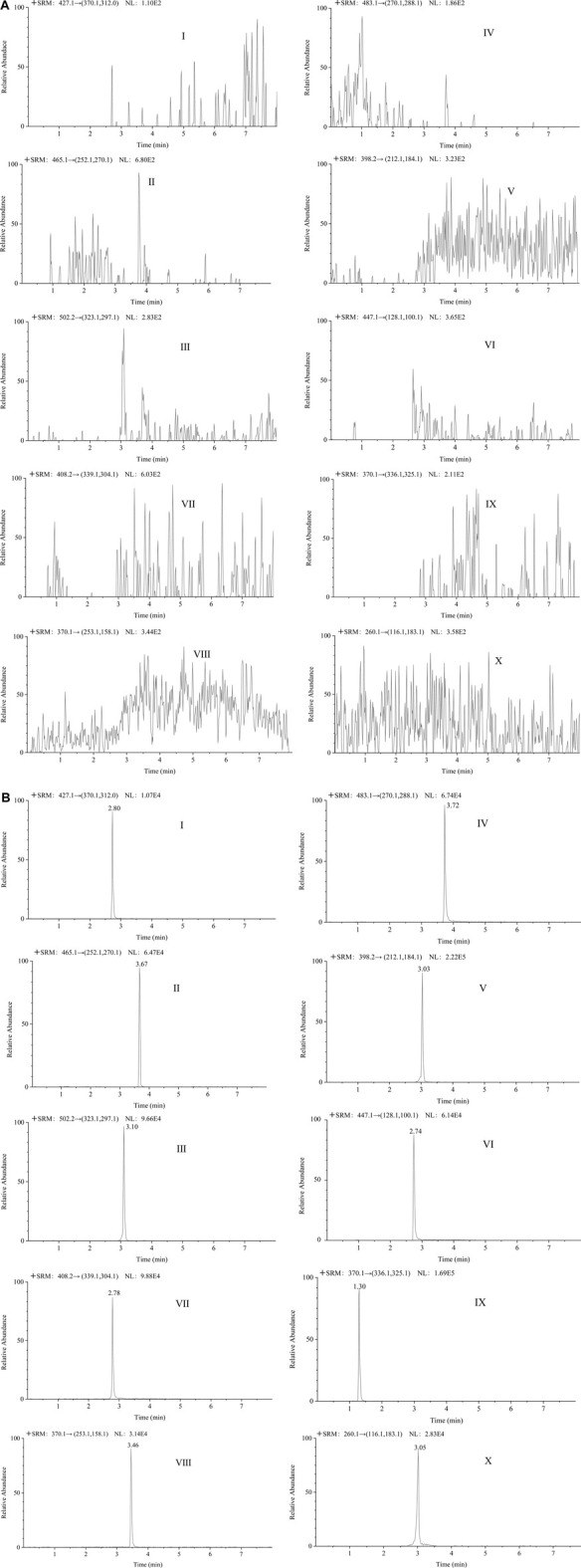
The representative chromatograms of lenvatinib **(Ⅰ)**, sorafenib **(Ⅱ)**, cabozantinib **(Ⅲ)**, regorafenib **(Ⅳ)**, apatinib **(Ⅴ)**, gefitinib **(Ⅵ)**, anlotinib **(Ⅶ)**, tivantinib **(Ⅷ)**, galunisertib **(Ⅸ)**, and IS **(Ⅹ)** in blank plasma samples **(A)**; each analyte in LLOQ samples **(B)**.

In the analysis of blank plasma samples, no interfering peaks from the endogenous substances were detected. Among these, the method was exclusively selective for the TKIs and IS. The representative chromatograms for the LLOQ and blank plasma samples are shown in Figure 8. The retention time for LEN, SOR, CBZ, RGF, APA, GEF, ANL, TIV, GAL and IS were 2.80, 3.67, 3.10, 3.72, 3.03, 2.74, 2.78, 3.46, 1.30, and 3.05 min, respectively.

#### 3.5.2 Calibration Curve and the Lower Limit of Quantification

The calibration curve of nine TKIs exhibited satisfactory linearity over the range of 0.1–10 ng/ml for lenvatinib, sorafenib, cabozantinib, apatinib, gefitinib, regorafenib, and anlotinib, and that of 1–100 ng/ml for tivantinib and galunisertib, while the linear correlation coefficients (R2) of all analytes was 0.9966–0.9999. The linearity, LODs, and LOQs of the nine analytes are shown in Table 2. LODs and LOQs indicated the sensitivity of the assay, the final results showed that the LODs of these analyzed hepatic agents targeting antineoplastic drugs were 0.003–0.11 ng/ml and the LOQs as 0.01–0.37 ng/ml, respectively. Therefore, the method was determined to be sufficiently sensitive for application in quantitative analyses.

**TABLE 2:** Linear regression equations, LODs, and LOQs of all TKIs.

Analyte	Linear equation	Linear range (ng/ml)	R^2^	LODs (ng/ml)	LOQs (ng/ml)
LEN	Y = 0.7709x + 0.2815	0.1–10	0.9968	0.01	0.04
SOR	Y = 0.3739x + 0.1048	0.1–10	0.9996	0.006	0.02
CBZ	Y = 0.0672x + 0.0318	0.1–10	0.9999	0.01	0.04
RGF	Y = 0.4654x + 0.0492	0.1–10	0.9977	0.003	0.01
APA	Y = 1.2796x + 1.0863	0.1–10	0.9982	0.006	0.02
GEF	Y = 0.3259x + 0.0084	0.1–10	0.9973	0.003	0.01
ANL	Y = 0.1886x + 0.0353	0.1–10	0.9975	0.01	0.04
TIV	Y = 0.0618x + 0.0296	1.0–100	0.9966	0.11	0.37
GAL	Y = 0.2120x − 0.2406	1.0–100	0.9992	0.05	0.18

#### 3.5.3 Precision and Accuracy

The results of intra-day and inter-day precision and accuracy at different concentration levels are depicted in Table 3. The precision for all TKIs was < 9.09%, whereas the accuracy value was -7.34–6.64%. Thus, this assay was deemed suitable for the detection of these nine TKIs with satisfying accuracy and precision at different concentration levels (LQC, MQC, HQC, and LLOQ).

**TABLE 3:** Results for intra-day and inter-day precision (RSD) and accuracy (RE) of QC and LLOQ samples (*n* = 6).

Analyte	Nominal concentration (ng/ml)	Intra-day		Inter-day	
		RSD (%)	RE (%)	RSD (%)	RE (%)
LEN	0.1	7.61	−3.83	8.23	5.85
	0.2	6.57	2.08	7.02	−6.45
	5	4.59	−6.44	5.67	3.65
	10	2.43	4.34	4.21	1.43
SOR	0.1	6.11	−4.43	7.41	4.78
	0.2	5.87	−3.32	7.76	−4.32
	5	2.96	1.70	6.95	5.77
	10	1.45	3.82	2.64	0.97
CBZ	0.1	7.21	4.34	9.09	−7.32
	0.2	6.74	3.90	7.08	3.98
	5	5.67	−1.32	5.28	4.76
	10	3.43	3.31	6.87	3.82
RGF	0.1	5.43	−1.32	8.23	1.34
	0.2	3.08	2.47	6.65	−7.32
	5	3.56	3.95	6.77	2.67
	10	1.22	−2.45	4.01	1.34
APA	0.1	5.35	−6.21	4.34	5.43
	0.2	6.65	4.67	6.64	4.77
	5	5.41	−7.34	5.63	4.98
	10	0.99	3.36	2.81	2.08
GEF	0.1	3.36	−2.24	5.44	2.96
	0.2	1.95	−2.94	2.78	1.77
	5	2.45	−1.95	1.46	2.04
	10	0.87	3.95	2.82	−4.55
ANL	0.1	5.34	4.27	8.45	5.54
	0.2	4.79	3.39	6.78	2.98
	5	3.47	−3.67	6.48	3.01
	10	3.46	6.64	5.73	3.83
TIV	1	6.46	5.99	3.97	−0.78
	2	4.38	2.63	5.56	1.15
	50	1.06	−2.27	2.58	2.97
	100	0.40	−3.82	2.20	1.04
GAL	1	3.36	3.67	3.82	−0.77
	2	4.02	0.30	6.92	5.44
	50	0.92	−2.01	3.02	−2.24
	100	1.29	4.88	2.24	−0.95

#### 3.5.4 MEs and Recovery

The recovery outcomes and MEs are depicted in Table 4. The recoveries of nine analytes at different concentrations ranged from 90.84 to 100.13%, and the RSD values were 1.85–9.29%. The recoverie of IS was 94.57%, and the RSD value was 6.86%. These results indicated a high extraction efficiency for nine TKIs. The MEs for all analytes ranged from 90.48 to 107.77% at the LQC and HQC levels, and the normalized matrix factors of the internal standard were determined by RSD to be < 9.74%. Therefore, the ME of the established method was negligible.

**TABLE 4:** Recovery and matrix effect of the 9 TKIs in the human plasma (*n* = 6).

Analyte	Concentration (ng/ml)	Recovery %		Matrix effect %	
		Mean	RSD	Mean	RSD
LEN	0.2	94.34	6.54	97.95	3.29
	5	97.72	5.32		
	10	96.82	3.91	99.54	1.91
SOR	0.2	93.83	4.25	102.76	6.24
	5	94.24	4.68		
	10	98.02	3.67	98.34	7.29
CBZ	0.2	96.92	2.73	106.46	5.32
	5	97.29	4.45		
	10	97.02	3.69	107.77	6.20
RGF	0.2	92.92	7.92	97.45	7.02
	5	90.84	5.74		
	10	93.02	5.31	95.88	3.91
APA	0.2	93.54	3.40	96.78	2.29
	5	100.13	3.73		
	10	98.39	2.44	99.34	3.65
GEF	0.2	96.28	5.40	105.29	5.72
	5	93.14	2.71		
	10	97.62	1.85	100.36	8.54
ANL	0.2	92.82	7.52	93.77	2.98
	5	98.94	4.28		
	10	96.02	3.61	98.64	4.92
TIV	2	91.64	9.29	90.48	2.23
	50	92.82	7.42		
	100	98.76	2.24	94.32	5.44
GAL	2	93.39	4.67	97.56	5.42
	50	95.85	6.88		
	100	96.20	3.83	104.67	9.74
IS	5	94.57	6.86

#### 3.5.5 Stability

Table 5 depicts the stability outcomes of the nine TKIs under five storage conditions. The values of RSD for the stability test were < 12.83%, which is within the acceptance criteria, indicating that all TKIs have acceptable stability under different storage conditions.

**TABLE 5:** Stabilities of the LQC and HQC samples under different storage conditions (*n* = 6).

Analyte	Concentration (ng/ml)	Expressed % (RSD %)				
		25°C/24 h	4°C/48 h	Autosampler/72 h	3 freeze-Thaw cycles	-20°C/15 days
LEN	0.2	93.38 (7.84)	96.38 (8.63)	96.76 (5.82)	93.24 (11.76)	89.23 (4.23)
	10	97.93 (3.88)	93.86 (4.71)	105.72 (7.65)	95.96 (5.83)	96.37 (6.34)
SOR	0.2	92.83 (6.39)	94.44 (10.67)	100.48 (8.73)	96.62 (6.03)	91.04 (8.04)
	10	104.22 (5.02)	103.26 (5.98)	106.27 (6.78)	97.28 (8.34)	103.75 (6.39)
CBZ	0.2	106.23 (0.89)	102.68 (8.19)	107.41 (10.54)	105.82 (4.73)	100.84 (7.33)
	10	109.02 (3.91)	103.28 (10.60)	108.19 (4.36)	106.25 (7.65)	110.45 (2.08)
RGF	0.2	78.20 (8.92)	87.55 (6.83)	92.56 (6.45)	87.06 (4.09)	76.87 (6.94)
	10	94.88 (6.20)	95.46 (1.82)	96.17 (8.32)	93.28 (5.86)	90.34 (5.98)
APA	0.2	91.05 (12.83)	93.53 (5.92)	96.35 (7.82)	92.43 (6.45)	89.43 (10.47)
	10	103.81 (9.54)	92.58 (3.86)	108.48 (10.27)	93.65 (5.02)	88.28 (8.22)
GEF	0.2	76.87 (2.98)	85.46 (1.38)	88.29 (3.67)	85.66 (5.45)	75.77 (6.38)
	10	87.17 (6.82)	81.25 (6.45)	96.23 (4.65)	88.54 (8.45)	80.65 (1.67)
ANL	0.2	109.55 (1.93)	107.47 (3.87)	111.87 (2.78)	102.68 (6.88)	98.63 (7.45)
	10	100.76 (6.55)	103.17 (8.56)	102.34 (5.85)	104.95 (7.85)	97.46 (9.43)
TIV	2	91.64 (5.92)	89.24 (12.62)	97.16 (11.02)	92.66 (9.44)	96.49 (8.95)
	100	95.76 (5.11)	92.48 (7.38)	99.35 (4.45)	89.54 (2.56)	85.34 (6.44)
GAL	2	104.28 (2.96)	100.66 (6.49)	106.28 (5.34)	95.32 (3.76)	97.56 (1.76)
	100	107.98 (3.81)	98.86 (4.78)	109.45 (2.64)	99.84 (4.87)	96.47 (7.56)

### 3.6 Comparative Analyses With Other Published Methods

This method was compared with other published assays for the TKIs with anti-hepatocellular carcinogenic effects in terms of LOQs and recovery (Table 6). The LC-MS/MS method revealed a large dynamic range and high sensitivity, and it is hence the most widely used detection method in the present literature. Among the pretreatment methods tested, the most commonly used methods included PP and LLE. The optimized QuEChERS method employed in this research was compared with other assays to reveal relatively and significantly better recovery and LOQs. Although the recovery rates of particular methods were similar, the LOQs were low. Therefore, finally, the QuEChERS method was used for further analyses considering that it is simple, efficient, and suitable for detection.

**TABLE 6:** Comparison of the proposed method with other published methods for the quantitative detection of TKIs with anti-hepatocellular carcinogenic effects.

Sample Type	Analyte	Detection system	Method	LOQ (ng/ml)	Recovery (%)	Ref
Plasma	9 TKIs	UPLC–MS/MS	QuEChERS	0.01∼0.37	90.84∼100.13	this work
Plasma	Cabozantinib	LC–MS/MS	PP	25	86.9 ± 5.4	Ferrer et al., (2020)
Serum/plasma	10 TKIs	LC–MS/MS	PP	2–6	89.5–110	Aghai et al., (2021)
Plasma	7 TKIs	UPLC–MS/MS	LLE	5	≥69	Ezzeldin et al., (2020)
Plasma	Galunisertib	LC–MS/MS	PP	0.05	85.96 ± 5.8	Tibben et al., (2019)
Plasma	6 TKIs	HPLC–Q–Orbitrap MS	LLE	0.02–2	88.3–103.4	Ni et al., (2017)
Plasma	7 TKIs and metabolite regorafenib M2	UPLC–MS/MS	PP	6–4,998.7	>70	Krens et al., (2020)
Plasma	sorafenib, regorafenib and their metabolites	LC–MS/MS	PP	30∼50	≥85.5	Iacuzzi et al., (2020)
Plasma	Lenvatinib	UPLC–MS/MS	SLE	0.2	98.63 ± 4.55	Sueshige et al., (2021)
Plasma	sorafenib, lenvatinib, and apatinib	UPLC–MS/MS	PP	1.3–312.5	90.5–99.4	Ye et al., (2021)
Plasma	apatinib and metabolites	UPLC–MS/MS	LLE	1	48.9–69.5	Guan et al. (2019)
Plasma	12 TKIs	LC–MS/MS	SALLE	0.5–12.5	83.19–112.04	Zhou et al. (2021)
Dried blood spots	Gefitinib	LC–MS/MS	PP	40	95.7–104.9	Irie et al. (2018)

### 3.7 Method Application

**FIGURE 9:**
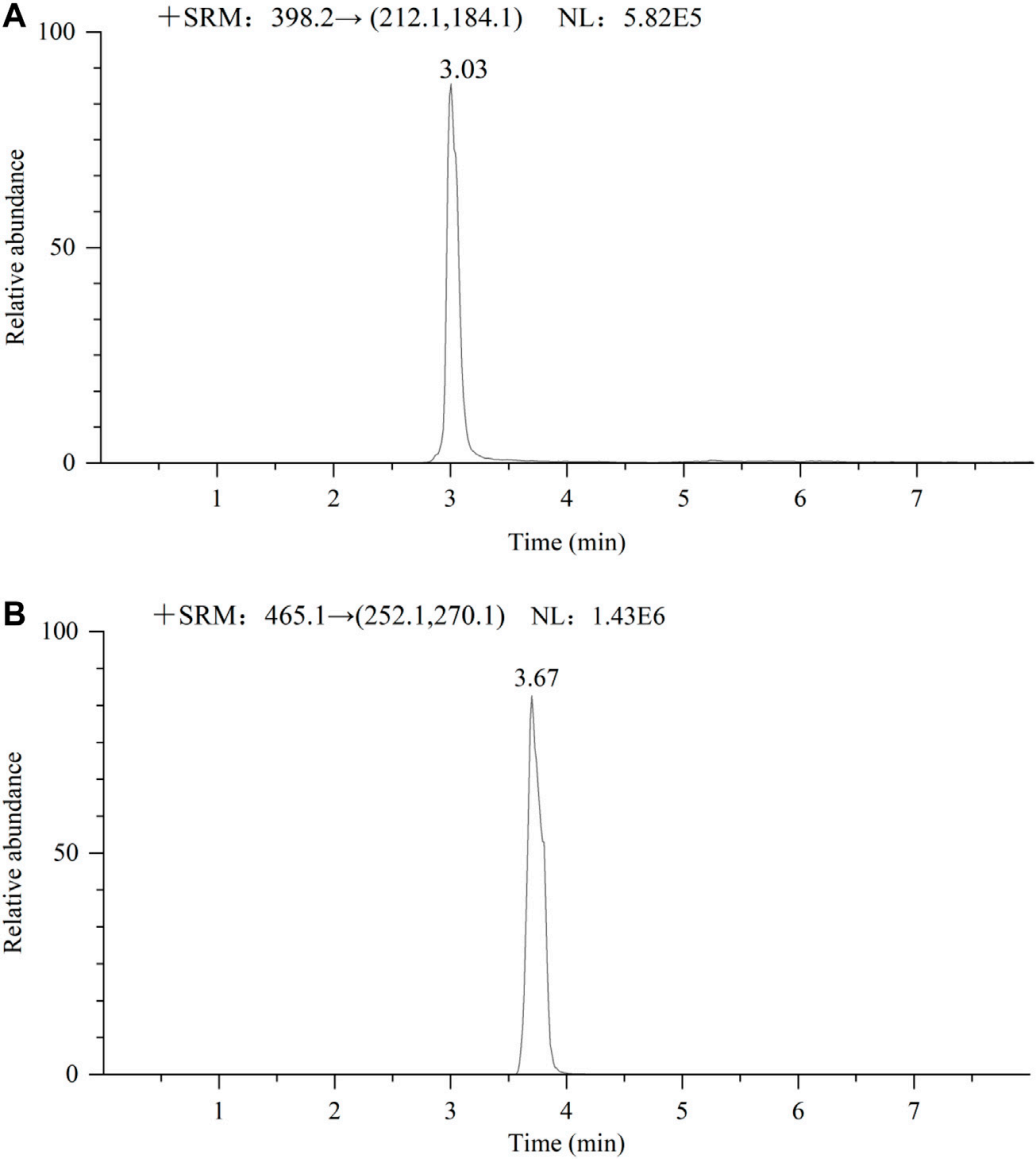
Chromatogram of 2 male patients receiving oral apatinib **(A)** 250 mg and sorafenib **(B)** 400 mg respectively.

This method is currently used only for blood concentration monitoring of apatinib and sorafenib because blood from patients with HCC is more difficult to obtain. The method was validated and has been successfully applied to the quantitative analysis of apatinib and sorafenib in the plasma of patients with HCC. The basic information of the two patients is as follows: sample 1 (male, age: 69 years), taking apatinib 250 mg daily; Sample 2 (male, age: 62 years), taking sorafenib 400 mg daily. Figures 9A,B show the chromatograms of apatinib and sorafenib in the plasma of patients with HCC, respectively. The blood drug concentrations were calculated to be 327 ng/ml and 3,842 ng/ml, respectively. The results suggest that the method is suitable for the detection of the nine TKIs in the plasma of patients with HCC.

## 4 Conclusion

In conclusion, in this study, we established a new method that combines the QuEChERS pretreatment technology with UPLC-MS/MS for the quantitative determination of nine TKIs in human plasma specimens. Accordingly, the factors of chromatographic conditions, MS conditions, and the QuEChERS method were optimized. When compared with the other available methods, the optimized method exhibited the advantages of simplicity, reliability, and rapidity. The LOQs of this method were 0.01–0.37 ng/ml and the total chromatographic run time was 8 min for each analyte. Moreover, the recovery and precision were found to be excellent, and the TKI samples showed acceptable stability under different conditions with negligible ME. Therefore, we recommend the proposed method for use in the routine quantitative assay to evaluate nine TKIs in the human plasma.

## Data Availability

The original contributions presented in the study are included in the article/supplementary materials, further inquiries can be directed to the corresponding authors.
